# Supplementary material for: The effects of genital myiasis on the diversity of the vaginal microbiota in female Bactrian camels
Source: BMC Vet Res. 2022 Mar 5;18:87. doi: 10.1186/s12917-022-03189-5 (PMC8897907; doi:10.1186/s12917-022-03189-5)
Supplement: Supplementary file 5 — Additional file 5. [file 12917_2022_3189_MOESM5_ESM.zip › MPL201709200_16s_yy/Treat1/css/js_css/jsPDF-master.zip!/jsPDF-master/docs/plugins_annotations.js.html]

plugins/annotations.js - Documentation


## Home

### Classes

- jsPDF

### Global

- addFont
- addHTML
- addMetadata
- addPage
- autoPrint
- CapJoinStyles
- circle
- ellipse
- getFontList
- lines
- lstext
- output
- rect
- roundedRect
- save
- setDisplayMode
- setDrawColor
- setFillColor
- setFont
- setFontSize
- setFontStyle
- setLineCap
- setLineJoin
- setLineWidth
- setPage
- setProperties
- setTextColor
- text
- triangle

# plugins/annotations.js

```
/**
 * jsPDF Annotations PlugIn
 * Copyright (c) 2014 Steven Spungin (TwelveTone LLC)  steven@twelvetone.tv
 *
 * Licensed under the MIT License.
 * http://opensource.org/licenses/mit-license
 */

/**
 * There are many types of annotations in a PDF document. Annotations are placed
 * on a page at a particular location. They are not 'attached' to an object.
 * <br />
 * This plugin current supports <br />
 * <li> Goto Page (set pageNumber and top in options)
 * <li> Goto Name (set name and top in options)
 * <li> Goto URL (set url in options)
 * <p>
 * 	The destination magnification factor can also be specified when goto is a page number or a named destination. (see documentation below)
 *  (set magFactor in options).  XYZ is the default.
 * </p>
 * <p>
 *  Links, Text, Popup, and FreeText are supported.
 * </p>
 * <p>
 * Options In PDF spec Not Implemented Yet
 * <li> link border
 * <li> named target
 * <li> page coordinates
 * <li> destination page scaling and layout
 * <li> actions other than URL and GotoPage
 * <li> background / hover actions
 * </p>
 */

/*
    Destination Magnification Factors
    See PDF 1.3 Page 386 for meanings and options

    [supported]
	XYZ (options; left top zoom)
	Fit (no options)
	FitH (options: top)
	FitV (options: left)

	[not supported]
	FitR
	FitB
	FitBH
	FitBV
 */

(function(jsPDFAPI) {
	'use strict';

	var annotationPlugin = {

		/**
		 * An array of arrays, indexed by <em>pageNumber</em>.
		 */
		annotations : [],

		f2 : function(number) {
			return number.toFixed(2);
		},

		notEmpty : function(obj) {
			if (typeof obj != 'undefined') {
				if (obj != '') {
					return true;
				}
			}
		}
	};

	jsPDF.API.annotationPlugin = annotationPlugin;

	jsPDF.API.events.push([ 'addPage', function(info) {
		this.annotationPlugin.annotations[info.pageNumber] = [];
	} ]);

	jsPDFAPI.events.push([ 'putPage', function(info) {
		//TODO store annotations in pageContext so reorder/remove will not affect them.
		var pageAnnos = this.annotationPlugin.annotations[info.pageNumber];

		var found = false;
		for (var a = 0; a < pageAnnos.length && !found; a++) {
			var anno = pageAnnos[a];
			switch (anno.type) {
			case 'link':
				if (annotationPlugin.notEmpty(anno.options.url) || annotationPlugin.notEmpty(anno.options.pageNumber)) {
					found = true;
					break;
				}
            case 'reference':
			case 'text':
			case 'freetext':
				found = true;
				break;
			}
		}
		if (found == false) {
			return;
		}

		this.internal.write("/Annots [");
		var f2 = this.annotationPlugin.f2;
		var k = this.internal.scaleFactor;
		var pageHeight = this.internal.pageSize.height;
		var pageInfo = this.internal.getPageInfo(info.pageNumber);
		for (var a = 0; a < pageAnnos.length; a++) {
			var anno = pageAnnos[a];

			switch (anno.type) {
            case 'reference':
                // References to Widget Anotations (for AcroForm Fields)
                this.internal.write(' ' + anno.object.objId + ' 0 R ');
				break;
			case 'text':
				// Create a an object for both the text and the popup
				var objText = this.internal.newAdditionalObject();
				var objPopup = this.internal.newAdditionalObject();

				var title = anno.title || 'Note';
				var rect = "/Rect [" + f2(anno.bounds.x * k) + " " + f2(pageHeight - (anno.bounds.y + anno.bounds.h) * k) + " " + f2((anno.bounds.x + anno.bounds.w) * k) + " " + f2((pageHeight - anno.bounds.y) * k) + "] ";
				line = '<</Type /Annot /Subtype /' + 'Text' + ' ' + rect + '/Contents (' + anno.contents + ')';
				line += ' /Popup ' + objPopup.objId + " 0 R";
				line += ' /P ' + pageInfo.objId + " 0 R";
				line += ' /T (' + title + ') >>';
				objText.content = line;

				var parent = objText.objId + ' 0 R';
				var popoff = 30;
				var rect = "/Rect [" + f2((anno.bounds.x + popoff) * k) + " " + f2(pageHeight - (anno.bounds.y + anno.bounds.h) * k) + " " + f2((anno.bounds.x + anno.bounds.w + popoff) * k) + " " + f2((pageHeight - anno.bounds.y) * k) + "] ";
				//var rect2 = "/Rect [" + f2(anno.bounds.x * k) + " " + f2((pageHeight - anno.bounds.y) * k) + " " + f2(anno.bounds.x + anno.bounds.w * k) + " " + f2(pageHeight - (anno.bounds.y + anno.bounds.h) * k) + "] ";
				line = '<</Type /Annot /Subtype /' + 'Popup' + ' ' + rect + ' /Parent ' + parent;
				if (anno.open) {
					line += ' /Open true';
				}
				line += ' >>';
				objPopup.content = line;

				this.internal.write(objText.objId, '0 R', objPopup.objId, '0 R');

				break;
			case 'freetext':
				var rect = "/Rect [" + f2(anno.bounds.x * k) + " " + f2((pageHeight - anno.bounds.y) * k) + " " + f2(anno.bounds.x + anno.bounds.w * k) + " " + f2(pageHeight - (anno.bounds.y + anno.bounds.h) * k) + "] ";
				var color = anno.color || '#000000';
				line = '<</Type /Annot /Subtype /' + 'FreeText' + ' ' + rect + '/Contents (' + anno.contents + ')';
				line += ' /DS(font: Helvetica,sans-serif 12.0pt; text-align:left; color:#' + color + ')';
				line += ' /Border [0 0 0]';
				line += ' >>';
				this.internal.write(line);
				break;
			case 'link':
				if (anno.options.name) {
					var loc = this.annotations._nameMap[anno.options.name];
					anno.options.pageNumber = loc.page;
					anno.options.top = loc.y;
				} else {
					if (!anno.options.top) {
						anno.options.top = 0;
					}
				}

				//var pageHeight = this.internal.pageSize.height * this.internal.scaleFactor;
				var rect = "/Rect [" + f2(anno.x * k) + " " + f2((pageHeight - anno.y) * k) + " " + f2(anno.x + anno.w * k) + " " + f2(pageHeight - (anno.y + anno.h) * k) + "] ";

				var line = '';
				if (anno.options.url) {
					line = '<</Type /Annot /Subtype /Link ' + rect + '/Border [0 0 0] /A <</S /URI /URI (' + anno.options.url + ') >>';
				} else if (anno.options.pageNumber) {
					// first page is 0
					var info = this.internal.getPageInfo(anno.options.pageNumber);
					line = '<</Type /Annot /Subtype /Link ' + rect + '/Border [0 0 0] /Dest [' + info.objId + " 0 R";
					anno.options.magFactor = anno.options.magFactor || "XYZ";
					switch (anno.options.magFactor) {
					case 'Fit':
						line += ' /Fit]';
						break;
					case 'FitH':
						//anno.options.top = anno.options.top || f2(pageHeight * k);
						line += ' /FitH ' + anno.options.top + ']';
						break;
					case 'FitV':
						anno.options.left = anno.options.left || 0;
						line += ' /FitV ' + anno.options.left + ']';
						break;
					case 'XYZ':
					default:
						var top = f2((pageHeight - anno.options.top) * k);// || f2(pageHeight * k);
						anno.options.left = anno.options.left || 0;
						// 0 or null zoom will not change zoom factor
						if (typeof anno.options.zoom === 'undefined') {
							anno.options.zoom = 0;
						}
						line += ' /XYZ ' + anno.options.left + ' ' + top + ' ' + anno.options.zoom + ']';
						break;
					}
				} else {
					// TODO error - should not be here
				}
				if (line != '') {
					line += " >>";
					this.internal.write(line);
				}
				break;
			}

		}
		this.internal.write("]");
	} ]);

	jsPDFAPI.createAnnotation = function(options) {
		switch (options.type) {
		case 'link':
			this.link(options.bounds.x, options.bounds.y, options.bounds.w, options.bounds.h, options);
			break;
		case 'text':
		case 'freetext':
			this.annotationPlugin.annotations[this.internal.getCurrentPageInfo().pageNumber].push(options);
			break;
		}
	}

	/**
	 * valid options
	 * <li> pageNumber or url [required]
	 * <p>If pageNumber is specified, top and zoom may also be specified</p>
	 */
	jsPDFAPI.link = function(x,y,w,h,options) {
		'use strict';
		this.annotationPlugin.annotations[this.internal.getCurrentPageInfo().pageNumber].push({
			x : x,
			y : y,
			w : w,
			h : h,
			options : options,
			type : 'link'
		});
	};

	/**
	 * valid options
	 * <li> pageNumber or url [required]
	 * <p>If pageNumber is specified, top and zoom may also be specified</p>
	 */
	jsPDFAPI.link = function(x,y,w,h,options) {
		'use strict';
		this.annotationPlugin.annotations[this.internal.getCurrentPageInfo().pageNumber].push({
			x : x,
			y : y,
			w : w,
			h : h,
			options : options,
			type : 'link'
		});
	};

	/**
	 * Currently only supports single line text.
	 * Returns the width of the text/link
	 */
	jsPDFAPI.textWithLink = function(text,x,y,options) {
		'use strict';
		var width = this.getTextWidth(text);
		var height = this.internal.getLineHeight();
		this.text(text, x, y);
		//TODO We really need the text baseline height to do this correctly.
		// Or ability to draw text on top, bottom, center, or baseline.
		y += height * .2;
		this.link(x, y - height, width, height, options);
		return width;
	};

	//TODO move into external library
	jsPDFAPI.getTextWidth = function(text) {
		'use strict';
		var fontSize = this.internal.getFontSize();
		var txtWidth = this.getStringUnitWidth(text) * fontSize / this.internal.scaleFactor;
		return txtWidth;
	};

	//TODO move into external library
	jsPDFAPI.getLineHeight = function() {
		return this.internal.getLineHeight();
	};

	return this;

})(jsPDF.API);
```

  

Documentation generated by JSDoc 3.4.2 on Sun Oct 09 2016 11:08:27 GMT+0100 (BST) using the docdash theme.
